# Cancer Cell Membrane-Coated Nanoparticles for Cancer Management

**DOI:** 10.3390/cancers11121836

**Published:** 2019-11-21

**Authors:** Jenna C. Harris, Mackenzie A. Scully, Emily S. Day

**Affiliations:** 1 Materials Science and Engineering, University of Delaware, Newark, DE 19716, USA; harrisj@udel.edu; 2 Biomedical Engineering, University of Delaware, Newark, DE 19716, USA; mkscully@udel.edu; 3 Helen F. Graham Cancer Center and Research Institute, Newark, DE 19713, USA

**Keywords:** biomimetic, nanocarrier, membrane-wrapped, cancer, targeted delivery, drug delivery, immunotherapy, photothermal therapy, photodynamic therapy, imaging

## Abstract

Cancer is a global health problem in need of transformative treatment solutions for improved patient outcomes. Many conventional treatments prove ineffective and produce undesirable side effects because they are incapable of targeting only cancer cells within tumors and metastases post administration. There is a desperate need for targeted therapies that can maximize treatment success and minimize toxicity. Nanoparticles (NPs) with tunable physicochemical properties have potential to meet the need for high precision cancer therapies. At the forefront of nanomedicine is biomimetic nanotechnology, which hides NPs from the immune system and provides superior targeting capabilities by cloaking NPs in cell-derived membranes. Cancer cell membranes expressing “markers of self” and “self-recognition molecules” can be removed from cancer cells and wrapped around a variety of NPs, providing homotypic targeting and circumventing the challenge of synthetically replicating natural cell surfaces. Compared to unwrapped NPs, cancer cell membrane-wrapped NPs (CCNPs) provide reduced accumulation in healthy tissues and higher accumulation in tumors and metastases. The unique biointerfacing capabilities of CCNPs enable their use as targeted nanovehicles for enhanced drug delivery, localized phototherapy, intensified imaging, or more potent immunotherapy. This review summarizes the state-of-the-art in CCNP technology and provides insight to the path forward for clinical implementation.

## 1. Introduction to Cancer and Nanomedicine

Cancer is a devastating global public health problem in desperate need of transformative solutions. It is the second leading cause of death in the United States and predicted to take 1700 lives per day in 2019 [1]. There is approximately a 37% chance a person will be diagnosed with cancer in their lifetime [1]. These alarming statistics indicate a critical need for technologies that can improve the early diagnosis and effective treatment of cancer. 

Conventional methods to treat cancer involve the surgical resection of tumors followed or preceded by aggressive chemotherapy and localized radiotherapy [2,3]. However, if tumors are non-resectable or metastasized, chemotherapy is the only therapeutic resolution to attempt to control the size and spread of the cancer [3]. Despite being the main clinical strategy, cytotoxic chemotherapeutics are incapable of targeting only cancer cells post-systemic administration [3,4]. Only a small fraction of drugs will accumulate in the desired tumor regions and metastatic lesions before being cleared from the body or entering non-targeted tissues [2,3,5]. Consequently, adverse side effects to healthy tissues limit the dosage of free drugs that can be administered, which reduces efficacy [6,7]. Further, due to the heterogeneous nature of tumors, which contain multiple cellular phenotypes, clinical practice and exploratory studies have shown that treatment regimens that employ only a single therapeutic agent are incapable of eliminating whole tumors and are even less effective in reducing and preventing metastasis [6]. There is a need for multimodal and synergistic cancer therapies that can improve patient outcomes [6,7]. 

Nanotechnology offers the opportunity to create nanovehicles that can carry either single or multiple therapeutic cargos, as well as contrast agents, to tumors for improved treatment and imaging. Nanoparticles (NPs) can be “smart designed” for enhanced drug delivery, phototherapy, vaccination, immunotherapy, and imaging [6,8]. Additionally, NPs can be synthesized with diverse physicochemical and surface properties that can be tailored to enhance cellular and molecular delivery, increase circulation times, facilitate crossing of biological barriers, and control cargo release [7,8,9]. Some nanomaterials can be designed with inherent optical or chemical properties that can be harnessed to enable stimuli-responsive therapy [10]. Nanovehicles can also be designed to integrate multiple therapeutic modalities in a single system to overcome the barriers experienced by cancer monotherapies [6].

The tumor microenvironment is characterized by leaky tumor vasculature and poor lymphatic drainage [3,8,9]. All systemically administered nanovehicles exploit this tumor pathophysiology to passively accumulate and be retained in tumor tissue; this is known as the enhanced permeability and retention (EPR) effect [4,11,12,13,14]. However, for NPs to utilize the EPR effect, they must first navigate the bloodstream, where they will be exposed to various proteins that may alter their surface chemistry. When NPs are coated with opsonin proteins, they are rapidly cleared by tissue resident macrophages of the liver and spleen, which limits their tumor delivery. For nanovehicles to efficiently enter tumors and be effective, they must evade detection by the immune system to exhibit long circulation, and protect their cargo from degradation or premature release [3,4,15]. Historically, NPs have been decorated with surface modifications, such as polyethylene glycol (PEG), to decrease their rapid opsonization and phagocytosis and increase their anti-tumor efficacy [3,4,7,16,17,18,19]. However, PEG-functionalized NPs can induce an “anti-PEG” immunological response and PEG does not impart NPs with cell-specific binding capabilities [3,16]. Additionally, PEGylated NPs are still cleared from the body, necessitating the use of more diverse and effective coatings. Researchers have coated NPs with ligands designed to enhance their cell-specific internalization via receptor-mediated processes to increase tumor retention and reduce off-target effects [17,18,20,21]. Still, there is substantial room for improvement. 

While ligand-targeted NP delivery to desired tumor cells is often depicted as a straightforward and easily accomplished task, it is extremely challenging to achieve this goal [21]. In part, this is due to the immense diversity in the abundance, variety, and complexity of proteins found on cancer cell membranes that might be targeted by NPs [3,10,22]. In addition to choosing the right molecule or combination of molecules to target, researchers must also carefully select the conjugation chemistry for ligand attachment to NPs [17,23]. Ligands that are too densely packed on an NP surface can cause a non-cooperative effect on target receptor binding, increased uptake by immune cells, and nonspecific binding to perivascular cells after extravasation [21,23,24]. This limits the NPs’ success due to low circulation time, early clearance from the body, and unwanted immune responses [16,18,19]. Further, serum proteins and opsonins can quickly coat ligand-targeted NPs in the bloodstream, rendering the targeting agents ineffective and increasing the rate of NP clearance from the body. These shortcomings create a need for surface modifications that can better disguise nanovehicles from the immune system, prolong circulation time, and provide enhanced targeting and cell internalization capabilities. 

**Figure 1:**
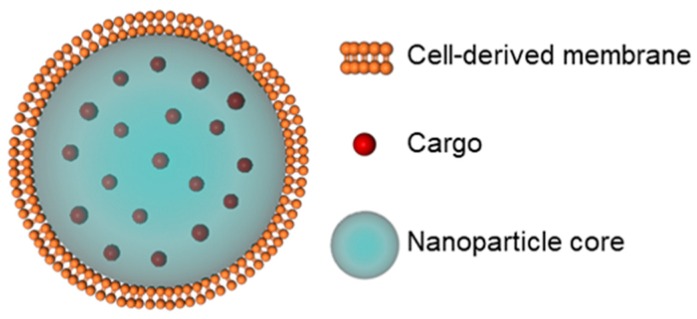
Scheme depicting the components of a representative membrane-wrapped nanoparticle.

Biomimetic nanotechnology harnesses the unique biological makeup of cell membranes and combines it with the flexibility of NP substrates and a wide range of payloads to improve targeted delivery. The general concept is to wrap NPs with cell-derived membranes that provide the complex biological entities found on natural cell membranes (Figure 1), which are nearly impossible to synthetically replicate via ligand attachments [22,25,26]. Since cell membranes contain both “markers of self” and “self-recognition molecules”, NPs wrapped in cell membranes can avoid immune recognition to maximally accumulate in tumors. Cell membrane coating technology was first introduced as a method to prolong NP circulation by using red blood cell (RBC) membranes to provide “stealth” properties to synthetic NPs [27]. It was shown that RBC-coated NPs exhibited a circulation half-life of 39.6 hours, substantially improved versus the 15.8-hour half-life of PEGylated NPs [27]. The field of cell membrane coating nanotechnology has since exploded with variations on this design [15,28,29,30,31,32]. Currently, cell membrane coating technology has been applied with many cell types, including platelets, leukocytes, cancer cells, stem cells, and more [33,34,35,36,37,38,39]. Cell membrane coatings have also been wrapped around a variety of materials, ranging from polymers to metals, to enhance their biointerfacing capabilities [2,8,22,40]. The two main advantages obtained from cell membrane wrapping are (1) reduced nonspecific uptake and (2) higher levels of specific targeting compared to non-wrapped NPs [2,25]. Notably, the hydrophobicity, charge, size, and structure of the core nanovehicles can be tailored to load desired cargoes within the interior without inhibiting the stealth or targeting properties of the membrane coating exterior [10]. Accordingly, cell membrane-coated NPs prove superior to previous NP synthesis techniques that have tried to reverse-engineer biological functions and interactions with limited success [3,4,41]. 

**Figure 2:**
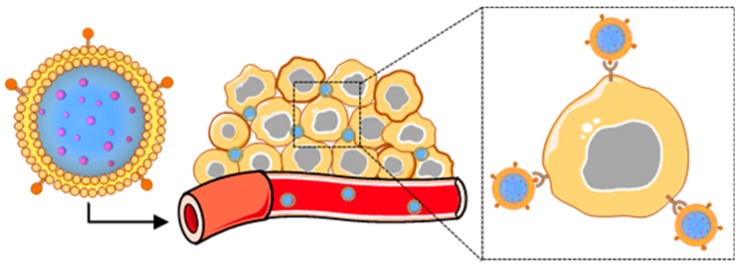
Scheme depicting the delivery of cancer cell membrane-wrapped nanoparticles (CCNPs) to tumors. Upon systemic administration, CCNPs exhibit long circulation due to the presence of “markers of self” on the membrane surface that minimize immune recognition. Additionally, CCNP membranes contain “self-recognition” molecules that allow the NPs to bind homotypic tumor cells after escaping from tumor vessels.

Cancer cell membranes are the ideal candidate to wrap around NPs for oncological applications [8,22]. Cancer cells are robust and easy to culture in large volumes in vitro for mass membrane collection and also possess the unique ability to self-target homologous cells (also known as homotypic targeting), unlike most other membrane donors [8,9,40,42]. This unique ability translates to cancer cell membrane-wrapped NPs (CCNPs), which retain the ability to homotypically target primary tumors and metastatic nodules [40,42,43,44,45] (Figure 2). Additionally, CCNPs display unprecedented binding and selective uptake in tumor cells matched to those from which they were derived, as well as have reduced immune clearance after systemic administration compared to non-coated NPs [22,40,42,44,46,47]. These unique properties enable CCNPs to be used as nanovehicles for enhanced chemotherapeutic drug delivery, localized phototherapy, intensified tumor imaging, or potent immune modulation.

In the following sections, we describe the synthesis and characterization of CCNPs and the different types of treatments these unique NPs can accomplish. We also provide a forward-looking perspective on the challenges to be addressed as this technology progresses from the laboratory setting to the clinic.

## 2. Cancer Cell Membrane-Wrapped Nanovehicles

### 2.1. Multi-step Synthesis of Cell Membrane-Wrapped Nanovehicles

**Figure 3:**
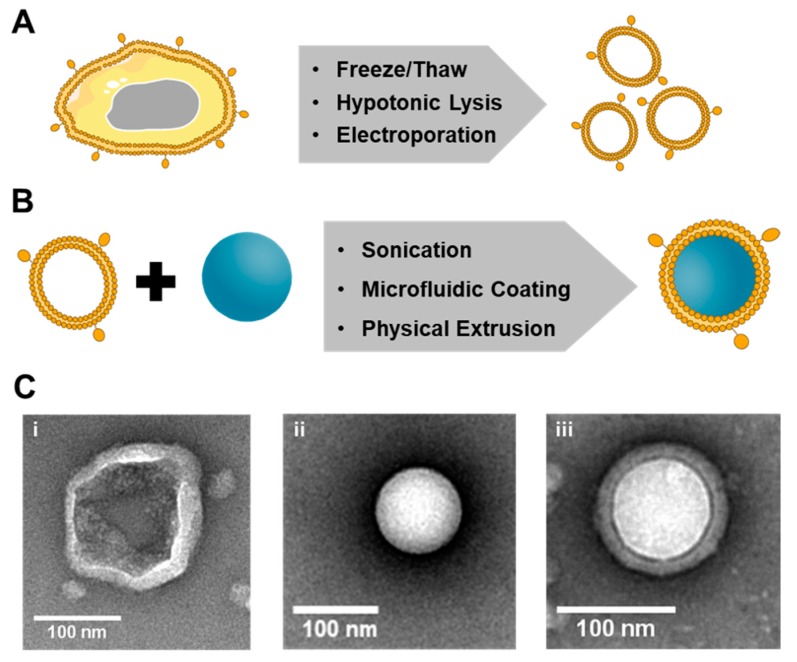
Illustration of the synthesis of membrane-wrapped nanoparticles. (**A**) Cell membranes can be extracted from their source cells by applying one of three methods. (**B**) Membranes can be wrapped around different types of nanoparticles using one of the three membrane–core fusion methods. (**C**) Transmission electron microscopy images of a (i) 4T1 breast cancer cell membrane vesicle, (ii) bare poly(lactic-co-glycolic acid) (PLGA) nanoparticle, and (iii) 4T1 cancer-cell membrane-wrapped PLGA nanoparticle prepared by the authors using the hypotonic lysis method depicted in (A) and the physical extrusion method depicted in (B).

The synthesis of cell membrane-coated nanovehicles involves three steps: (1) membrane extraction from source cells, (2) fabrication of the nanoparticulate core, and (3) fusion of the membranes and nanoparticulate cores to form core-shell membrane-wrapped NPs (Figure 3). Below, each step is described in detail.

#### 2.1.1. Membrane Extraction

At its most basic level, membrane extraction requires that internal cell components are removed while leaving the functional components of the membrane intact. This membrane extraction procedure requires large volumes of cells to be harvested from culture dishes or blood and tissue samples [8,22,31,48,49]. This process has been accomplished in many ways including freeze–thaw cycling [36,48,50], electroporation [51], and osmosis-based lysis coupled with physical homogenization [22,48] (Figure 3A). For freeze–thaw techniques, cells are frozen at −80 °C and thawed at either room temperature or 37 °C in repeated cycles. These cycles cause damage to cell membranes due to breakage of ice crystals, which leads to the removal of the cytosol and retention of the membranes. This technique is most appropriate for non-nucleated cells, such as RBCs or platelets, since the freeze steps can potentially cause damage such as loss of membrane structure, reduced protein stability, and consequent protein unfolding and reduced membrane function [10,36,48]. 

Electroporation lyses cells by exposing them to strong electric fields, causing temporary loss of semi-permeability, and pore formation in the cell membrane, releasing intracellular components [51]. Consequently, electroporation disruption of membranes results in irreversible deterioration of structural integrity, denaturation of membrane proteins, and loss of lipid asymmetry. Therefore, care must be taken when designing the experimental set-up of electroporation as conditions that are too harsh can cause loss of natural membrane potential [51,52]. 

The most popular method to extract cancer cell membranes involves osmosis-based cell lysis with a mild hypotonic solution, followed by mechanical membrane disruption with a homogenizer [48]. Discontinuous gradient centrifugation removes intracellular biomacromolecules, intracellular vesicles, and nuclei, and the membrane-rich fraction is washed with isotonic buffers to obtain membrane vesicles [23,53]. These vesicles can then be further sonicated or extruded through polycarbonate membranes to produce vesicles of the desired size [48]. Cancer cells require milder lysis conditions and greater ultracentrifugation speeds compared to non-nucleated cells. The differences in osmosis-based membrane extraction methods deviate between cell types due to eukaryotic cells’ phospholipid bilayer fluidity and smaller cell size [10]. 

#### 2.1.2. Selection of Nanoparticle Core

**Figure 4:**
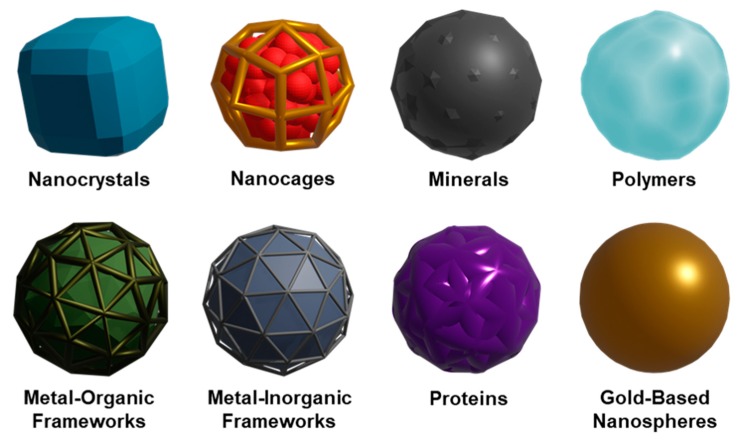
Summary of various nanoparticle formulations that have been wrapped with cell-derived membranes to enable cancer treatment and imaging.

As a variety of NP core designs may be utilized to produce CCNPs, depending on the intended application, it is unwarranted to describe any one specific NP synthesis here. The main criterion, independent of core material, is that the NPs have a negative zeta potential. This will facilitate proper orientation of the membrane around the NP owing to electrostatic repulsion between the NP surface and negative extracellular membrane components [27]. To date, the types of synthetic NPs that have been wrapped with cell-derived membranes for cancer therapies include nanocrystals [54], nanocages [42], mineral-based or mesoporous silica [35,49,55,56,57,58], polymeric cores [30,40,45,59,60,61,62,63,64], organic and inorganic metal frameworks [44,51,65,66,67], protein cores [68,69], and gold-based or magnetic nanoparticles [70,71,72] (Figure 4, Table 1). Poly(lactic-co-glycolic) acid (PLGA) is one of the most widely used NP cores due to its biodegradability, FDA approval, and ability to encapsulate many products [17,18,19]. Metallic-based NPs have also been widely used because they can aid in imaging and thus provide multiple functions [32,61,65,71,72]. Overall, the composition of the nanovehicle core is an important consideration when designing CCNPs as it dictates the release and efficacy of the cargo once it has been guided to the desired cells by the membrane coating.

#### 2.1.3. Fusion of Membrane Vesicles with Nanoparticle Cores

Methods of coating NPs with membranes can be divided into three generalized strategies: physical extrusion, sonication, and microfluidic coating (Figure 3B). All of these methods take advantage of electrostatic interactions between the nanoparticulate core and membrane components to form a stable and energetically favorable core-shell structure with the right-side-out membrane topological orientation [27,36,48]. In physical extrusion, nanovehicles and membrane vesicles are co-extruded through a porous membrane, similar to how membrane vesicles are formed by mechanical extrusion [27]. The force provided by the extrusion disrupts the membrane structure and enables it to reform around the NP cores [27,48]. A representative transmission electron micrograph of a CCNP prepared by extrusion in the authors’ lab is shown in Figure 3C. Here, the CCNP is composed of a PLGA core surrounded by a membrane derived from a 4T1 mouse breast cancer cell. In the authors’ experience, the extrusion method is very robust in terms of reproducibility and creating CCNPs with consistent characteristics (size, zeta potential, membrane thickness, etc.). In sonication-based methods, nanovehicles and membranes are again combined, and ultrasonic energy provides disruptive forces that result in spontaneous formation of core-shell nanostructures [53,59]. This technique has the added benefit of losing less material than physical extrusion. Lastly, a relatively new approach to enable membrane coating is to employ microfluidics. This fabrication technique combines rapid mixing of NPs and membrane vesicles with electroporation and has successfully been used to coat RBC membranes around magnetic NPs [51]. For this strategy to be successful, the process pulse voltage, duration, and flow velocity all have to be optimized, making it a potentially more difficult method to attempt for those not already familiar with microfluidics. 

### 2.2. Characterization of Membrane-Coated Nanoparticles

It is critical to compare various features between bare and wrapped NPs to confirm complete membrane wrapping. Successful wrapping can be validated by observing a 10–20 nanometer increase in particle size after wrapping, equating to the thickness of the membrane layer. This can be measured by dynamic light scattering (DLS), transmission electron microscopy (TEM) (Figure 3C), or nanoparticle tracking analysis (NTA). Analysis of the NPs’ zeta potential, or surface charge, can also be used to confirm membrane wrapping. The final charge of the CCNPs must be similar to that of the membrane vesicles used to prepare them, as the bare NPs inherit the surface charge of the membranes after successful wrapping. To further confirm successful membrane wrapping and the removal of intracellular components, Western blotting and SDS-PAGE can be performed to identify the main protein components of whole cell lysate, membrane lysate, and membrane-wrapped NPs. Membrane-wrapped NPs should share nearly identical protein content to the membrane lysate, but lack the nuclear and mitochondrial components of the whole cell lysate. Individual membrane surface markers can also be identified and their intensity compared between samples to confirm their successful translocation onto NPs from source cells during membrane wrapping. For example, Fang et al. showed by Western blotting that CCNPs prepared by physically extruding PLGA NPs with B16-F10 mouse melanoma membranes collected by hypotonic lysis were positive for the membrane markers pan-cadherin, Na^+^/K^+^-ATPase, and gp100, but lacked the intracellular markers histone H3, cytochrome c oxidase, and glyceraldehyde 3-phosphate dehydrogenase [45]. These data indicate that the preparation of CCNPs by hypotonic membrane lysis followed by physical extrusion with core NPs offers excellent preservation of the components of the original cell membrane. In the future, researchers utilizing other methods to prepare CCNPs should perform similar analyses to reveal which method imparts CCNPs with the greatest resemblance to their source cells.

## 3. Applications of Membrane-Wrapped Nanoparticles in Cancer

### 3.1. Drug Delivery

Cancer drug delivery is one field in which membrane-wrapped nanovehicles, and CCNPs in particular, have substantial potential to improve the state-of-the-art. Encapsulating drug cargo within nanocarriers that offer tailorable control of release kinetics, such as polymer-based cores, can dramatically improve bioavailability, and tumor-specific delivery can be further enhanced by coating these vehicles with cancer cell membranes [17,18,19]. Moreover, synthetic NPs’ physicochemical properties can be modified for sustainable or triggered cargo release, resulting in less systemic toxicity than freely delivered cargo [4,22,47,77]. As an extra benefit, membrane coatings can provide an additional decrease in premature drug release by slowing diffusion and allowing nanovehicles to accumulate in tumors before too much drug is lost [10,22]. Increasing the ratio of drug that reaches tumors versus normal tissue is critical to maximize therapeutic effects and safety. 

Doxorubicin (DOX) is a commonly used chemotherapeutic that intercalates into DNA to yield topoisomerase II-mediated DNA damage followed by cell death [78]. DOX has been used clinically to treat many cancers, including breast cancer, ovarian cancer, and various lymphomas and leukemias [78]. Several researchers have shown that encapsulating DOX in membrane-wrapped NPs is advantageous compared to freely delivered DOX [56,62,63]. For example, Xu et al. developed PLGA-DOX NPs wrapped in membranes derived from HepG2 hepatocarcinoma cells and showed these NPs could deliver an effective drug payload to Hep2G tumors in mice [62]. Additionally, the CCNPs exhibited less systemic toxicity than freely delivered DOX. This was attributed to enhanced DOX accumulation at the tumor site (and less accumulation at off-target sites) due to lack of premature release from the particles [62]. Another chemotherapeutic small molecule, paclitaxel (PTX), which is clinically used to treat AIDS-related Kaposi sarcoma, breast, non-small cell lung, and ovarian cancers [79,80,81,82,83,84,85], has been explored in conjugation with membrane-wrapped NPs. In one study, PTX was loaded into poly(caprolactone) (PCL) and pluronic copolymer F68 cores that were wrapped with 4T1 mouse mammary breast cancer cell membranes [40]. The homotypic targeting and drug delivery capabilities of these cancer cell membrane-wrapped PTX-loaded polymeric nanoparticles (CPPNs) were explored in a highly metastatic 4T1 in vivo tumor model. CPPNs remarkably targeted and inhibited the growth of homotypic 4T1 primary tumors and metastatic nodules in orthotopic mammary tumor models and in blood-vessel-metastasis mouse models, with 6.5-fold fewer metastatic nodules than unwrapped PPNs [40]. The intact 4T1 cell membrane wrapping decreased phagocytic uptake and increased blood-circulation time to increase the antitumor effect of the drug payload.

In addition to single drugs, multiple cargoes with synergistic actions can be encapsulated in CCNPs. This ensures the cargos are delivered to the same cells within tumor sites for improved anticancer effects. This was demonstrated with PLGA cores that were loaded with hemoglobin (Hb) and DOX and coated with MCF-7 human breast cancer cell membranes with a PEGylated phospholipid to overcome hypoxia-induced chemoresistance [63]. By suppressing the expression of hypoxia-inducible factor-1α, multidrug resistance gene 1, and P-glycoprotein, the biomimetic oxygen nanocarriers were able to perform safe and highly efficient O2-interfered chemotherapy by reducing the exocytosis of DOX. Simultaneously, the system achieved higher tumor specificity and lower DOX toxicity due to the cancer cell adhesion molecules retained on the NP surface [63]. This was an excellent demonstration of the potential for CCNPs to achieve multi-therapeutic delivery. In a similar approach, Chen et al. developed CCNPs to deliver DOX in combination with small interfering RNA (siRNA) against PD-L1, a gene that is overexpressed on tumor cells and whose inhibition could lead to an increased anti-tumor immune response [46]. Here, both cargos were loaded into PLGA NP cores and homotypic targeting was achieved by wrapping the NPs with HeLa cervical cancer cell membranes [46]. The CCNPs exhibited preferential uptake by HeLa cells versus non-targeted MDA-MB-231 breast cancer cells, and were able to suppress PD-L1 expression and reduce cell viability. Future studies are necessary to evaluate the impact of this system in vivo.

CCNPs that incorporate stimuli-responsive features have also been designed to take advantage of the acidic tumor microenvironment as a trigger for localized drug release [63]. In one example, mesoporous silica nanoparticle (MSN) cores were used to encapsulate DOX with the addition of a unique CaCO3 interlayer [56]. The interlayer acted as sheddable pH-sensitive gatekeeper to allow drug release only in the acidic tumor microenvironment. MSNs were wrapped with LNCaP-AI prostate cancer cell membranes (MSN/DOX@CaCO3@CM) to improve the colloidal stability and tumor accumulation of the system. In comparison to free DOX, MSN/DOX@CaCO3@CM NPs exhibited increased cell uptake and induced higher rates of apoptotic death in prostate cancer cells. In vivo experiments demonstrated that the NPs had remarkable antitumor effects and suppressed tumor growth [56]. Overall, this study demonstrated that coupling the increased localization of CCNPs in tumor microenvironments with pH-stimulated release of chemotherapeutic drugs is a potent strategy to enhance therapeutic ratios. 

Importantly, across various platforms, it has been shown that cancer cell membrane coatings do not negatively interfere with drug loading inside NPs. As demonstrated by the examples discussed, there is great promise in the field for CCNPs to enhance drug delivery to desired sites to improve safety and efficacy. This opens the door for the development of many new treatment strategies. 

### 3.2. Photothermal and Photodynamic Therapy

While some NPs exploit features of the tumor microenvironment such as low pH or presence of specific enzymes to enable stimuli-responsive drug release and high precision therapy, another route to enable site-specific treatment of tumors is to utilize nanomaterials that are inactive until they are triggered with externally applied light. The two main examples of this are photothermal therapy (PTT) and photodynamic therapy (PDT), and both have recently been explored in conjugation with membrane-wrapped NPs. In photothermal therapy, NPs with unique optical properties are delivered into tumors, which are then irradiated with near-infrared light that causes the NPs to produce heat capable of thermally damaging cancer cells [86,87,88,89,90,91,92]. Similarly, in PDT, photosensitizers are delivered into tumors, and subsequent irradiation of the tumor causes the photosensitizer to transfer the absorbed energy to adjacent tissue oxygen molecules, producing toxic singlet oxygen that destroys cancer cells [89]. While there are some examples of membrane-wrapped NPs being used strictly for PTT or PDT to treat cancer [70,93,94,95,96,97,98], these singular treatments use non-cancer cell membranes. When cancer cell membranes are used for wrapping, they are commonly studied in combination with other therapeutic strategies, such as drug delivery. Some accomplishments in this field are summarized below.

#### 3.2.1. Combination Photothermal Therapy and Chemotherapy

Combining PTT with chemotherapy offers many advantages versus either treatment alone. Several studies have shown that PTT can elevate drug delivery into tumors or into cancer cells by increasing vascular permeability and cancer cell membrane permeability [86,99]. Additionally, PTT alone is best suited for primary tumors, as it cannot be readily applied to disseminated metastatic tumors. Combining PTT with chemotherapy offers a way to treat both primary tumors and metastatic lesions. Further, there is some evidence that under the right conditions, combined PTT and chemotherapy can lead to anti-cancer immune responses that maximize the duration of response [100]. Given these advantages, researchers have explored the co-delivery of photothermal agents and cytotoxic drugs to cancer using CCNPs.

Combination PTT and chemotherapy mediated by CCNPs has been most widely explored using DOX as the chemotherapeutic agent [42,57,76]. In all cases, the DOX had improved tumor delivery due to the cancer cell membrane coating of the system and DOX was able to act successfully in combination with PTT to decrease tumor growth. In one study that co-loaded DOX and indocyanine green (ICG) photothermal agents in membrane-wrapped NPs, DOX was delivered in a “bomb-like” manner to the tumor surroundings [76]. This was due to the HeLa cervical cancer cell membrane wrapped around the cargo being disrupted by PTT, which led to enhanced chemo-PTT efficacy [76]. In an unusual case of using multiple types of membranes to coat NPs, RBC and B16-F10 mouse melanoma membranes were mixed to create a hybrid membrane that provided increased immune evasion and tumor targeting, respectively. The membranes were wrapped around DOX-loaded copper sulfide NPs, and these NPs exhibited synergistic effects with close to 100% tumor growth inhibition [57]. Lastly, when DOX was loaded into the core of gold nanocages, the hyperthermia induced-release of DOX in the targeted cells inhibited the growth of both primary tumors and metastatic nodules in a highly metastatic 4T1 mouse mammary tumor model [42]. These findings demonstrate the immense potential of combining PTT with chemotherapy using CCNPs.

#### 3.2.2. Photodynamic Therapy Combined with Chemotherapy or Starvation Therapy

The benefits of combining chemotherapy with PDT include having reactive oxygen species (ROS) available to initiate drug release and promote intracellular drug delivery, as well inducing hypoxia in the tumor region for activating encapsulated drugs [6]. Conversely, a limitation of PDT is that it relies on tumor oxygen, and is therefore not effective in hypoxic tumor regions. By combining PDT with drugs that are not hindered by hypoxia, more thorough tumor treatment can be achieved. In one example of dual PDT/chemotherapy, a porphyrinic metal organic framework (a PDT photosensitizer) was combined with tirapazamine (TPZ, a bioreactive chemotherapeutic) [73]. These agents were wrapped in membranes derived from 4T1 breast cancer cells, and the resultant NPs were delivered to mice bearing orthotopic 4T1 breast cancer tumors. Following irradiation, the porphyrinic metal organic frameworks produced ROS, leading to local hypoxia within the tumors, which accelerated the activation of TPZ for an enhanced chemotherapeutic effect. Because the treatment was activated only in the presence of light at the tumor site, negligible side effects were observed [73].

Besides being combined with chemotherapy, PDT has also been combined with starvation therapy. In starvation therapy, glucose oxidase (GOx) is delivered to tumors. GOx will transform glucose into gluconic acid and hydrogen peroxide, starving the cells of glucose, a vital nutrient for tumor growth. In one example, a cascade reaction system made of hollow manganese dioxide (MnO_2_) NPs encapsulating a photosensitizer and coated with GOx were wrapped in B16-F10 cancer cell membranes [67]. Once delivered to tumors, the MnO_2_ reactors were irradiated for continuous oxygen generation, supported by the conversion of glucose to singlet oxygen. This system has potential to solve hypoxia issues in tumors and promote starvation. Starvation therapy mediated by membrane-wrapped NPs has also been explored without PDT [55]. In this study, GOx-loaded membrane-wrapped mesoporous silica NPs were combined with PD-1 antibody treatment and shown to be more effective at stimulating an anti-cancer immune response than the single therapies, resulting in better cancer ablation. The above examples demonstrate that CCNPs have the ability to target tumor cells throughout the body and enable PTT or PDT in combination with chemotherapy or starvation therapy. These combinatorial delivery systems are more effective than monotherapies and offer extremely high precision treatment of tumors since they are activated only when light and NPs are combined at the tumor site. Continued development of these platforms will likely yield impressive results against a variety of tumor types.

### 3.3. Tumor Imaging

In many cases, it is desirable to monitor the accumulation of membrane-wrapped NPs within tumors, as this can guide and inform drug delivery, PDT, and PTT [44,61,72,74]. Most NP cores that enable imaging with high contrast are metallic-based, such as iron oxide or lanthanide-doped nanocrystals, but development of organic or polymer-based nanoparticles as imaging agents has also been explored [44,54,72,74].

**Figure 5:**
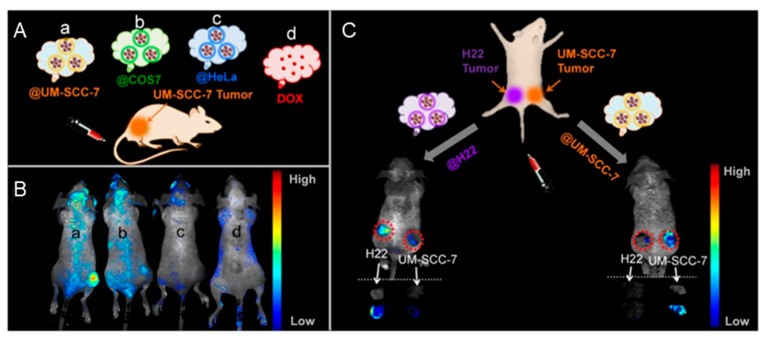
Demonstration of homotypic tumor targeting by CCNPs. (**A**) Illustration of experimental design for data shown in (**B**). Mice bearing human squamous carcinoma (UM-SCC-7) tumors were treated with doxorubicin (DOX) alone or with DOX and magnetic iron oxide nanoparticles that were wrapped with membranes derived from three different sources (COS7 monkey kidney cells, HeLa cervical cancer cells, or homotypic UM-SCC-7 squamous carcinoma cells). (**B**) In vivo fluorescence images of mice bearing UM-SCC-7 tumors 24 hours post-injection with membrane-wrapped nanoparticles prepared with (**a**) UM-SCC-7, (**b**) COS7, or (**c**) HeLa membranes as described in A, or post-injection with (**d**) DOX at an equivalent DOX dosage. The highest tumor accumulation is observed for homotypic membrane-wrapped nanoparticles. (**C**) Illustration of the dual tumor-bearing mouse model in which one flank harbored a hepatocellular carcinoma (H22) tumor and the other harbored a UM-SCC-7 tumor. The animals were injected with membrane-wrapped NPs designed to homotypically target one tumor or the other. Twelve hours post-injection, in vivo fluorescence images and ex vivo images of tumors were acquired. Both types of membrane-wrapped nanoparticles evaluated exhibited preferential accumulation in homotypic tumors (matched to the source membrane) versus heterotypic tumors with membrane mismatch. Reprinted (adapted) with permission from Reference [44]: Zhu, J.Y.; Zheng, D.W.; Zhang, M.K.; et al. *Nano Lett.* **2016**, *16*, 5895–5901. Copyright (2016) American Chemical Society.

In a study whose sole purpose was to view homologous targeting of upconversion nanoprobes (UCNPs), researchers used multiple types of cancer cell membranes (breast, prostate, colorectal, and squamous cell cancer) to prepare corresponding batches of wrapped lanthanide-doped nanocrystals [54]. These NPs could convert near-infrared (NIR) light into visible light, providing high signal-to-noise ratio. The specificity of homotypic membrane-mediated targeting was beautifully exhibited when mice bearing MDA-MB-435 breast cancer tumors were separately treated with each type of membrane-wrapped UCNPs, as only UCNPs wrapped in membranes derived from MDA-MB-435 cells exhibited notable tumor retention. This indicates that while the cancer-membrane-wrapped NPs possess the same immune evasion potential as RBC-wrapped NPs, mismatch of the donor membranes and host tumor cells leads to little tumor targeting. In a similar study, researchers developed magnetic iron oxide NPs loaded with DOX-HCl and coated them in either UM-SCC-7 squamous cell carcinoma or H22 hepatocellular carcinoma membranes [44]. The team used magnetic resonance imaging to show in mice bearing each type of tumor that the particles could bypass the heterologous tumor and preferentially target their homotypic tumor (Figure 5). Future research could evaluate the degree of mismatch that is acceptable when preparing CCNPs to maintain homotypic binding. While both of these studies used cells derived from different tumor types to demonstrate that homotypic binding requires membrane:tumor matching, it would be interesting to investigate if membranes derived from cancer cells that are from the same tissue but exhibit different biomarkers can provide targeted delivery.

Membrane-wrapped NPs that incorporate both contrast agents and photoactive agents have also been used for tumor imaging and phototherapies. In one study, ICG-loaded NPs wrapped with MCF-7-PEG fused membranes exhibited a PTT response, but also had fluorescence and photoacoustic (PA) imaging capabilities [74]. PEG incorporation in this system diminished non-specific binding of serum proteins and helped stop aggregation and opsonization leading to phagocytosis in vivo. This particle formulation ablated tumors after a single dose and laser irradiation, and provided high spatial resolution imaging of the tumor microstructure through PA imaging of the ICG signal in and around tumor microvesicles. In another study, MCF-7 membranes were fused with RBC membranes to coat melanin nanoparticles [68]. The biocompatible melanin core provided both PTT and PA imaging and the size dependence of the particles for optimal PTT and PA imaging were explored to find the balance between the two. 

Similarly, dual-modal imaging has been used with PDT by loading photosensitizers into magnetic nanobeads to target hepatocellular carcinoma [72]. Both near-infrared (NIR) fluorescence imaging and magnetic resonance (MR) imaging could be accomplished with this system, with NIR enabled by the loaded chlorin e6 (Ce6) photosensitizers that also provided PDT capabilities, and MR provided by the superparamagnetic iron oxide nanostructures. In an even more complex system, multimodal cancer phototheranostics were explored for the early diagnosis and precision therapy of cancer [61]. Here, organic, multimodal, NIR-semiconducting polymer NPs were produced, generating NIR and PA signals for imaging, as well as singlet oxygen and cytotoxic heat for combinatorial PDT-PTT effects. These NPs were coated in either activated fibroblast membranes or 4T1 cancer cell membranes, where interestingly, the fibroblast-coated NPs performed better than their cancer cell membrane-coated counterparts [61]. Although the 4T1 membrane NPs targeted cancer cells, they were limited by the abundance of cancer-associated fibroblasts in the tumor environments, making their accumulation only marginally higher than non-targeted particles. The activated fibroblast-coated NPs, in contrast, exhibited heightened tumor accumulation through their homotypic targeting of cancer-associated fibroblasts at the tumor site.

### 3.4. Immune Stimulation

There is growing interest in the field of oncology in using technologies to stimulate the body’s immune system to attack tumor cells. Immunotherapy can be applied as either a cancer treatment or as a preventative cancer vaccination, and is advantageous over cytotoxic agents because of its high specificity and low toxicity [2,6,18,75,101,102,103,104,105,106]. Effective immune stimulation typically requires that both adjuvants and tumor antigens be delivered to the body. One advantage of CCNPs is that their membrane coatings carry a full array of cancer cell membrane antigens that can stimulate an anticancer immune response. Thus, combining CCNPs with adjuvant technologies is a promising strategy to elicit robust anti-tumor responses. In the following sections, it is assumed readers have a basic familiarity with cancer immunotherapy. For detailed reviews, we refer the readers to recent publications [102,105,106,107,108,109,110].

Exploring the immune stimulatory capabilities of membrane-wrapped NPs, Fang et al. prepared PLGA NPs wrapped in B16-F10 melanoma or MDA-MB-435 human breast cancer membranes and utilized these NPs to deliver antigens to source tumor cells and antigen presenting cells (APCs) [45]. The CCNPs enhanced source cell-specific binding and uptake compared to RBC-wrapped NPs and bare NPs. They also successfully delivered membrane-bound tumor-associated antigens to APCs. However, this was insufficient to induce dendritic cell maturation due to the limited immunogenicity of syngeneic cancer cell membrane material. To overcome this issue, the team incorporated monophosphoryl lipid A (MPLA), an immunoadjuvant lipopolysaccharide derivative that binds to toll-like receptor 4 (TLR-4), into the CCNPs, and this significantly increased the APC maturation to promote an anticancer immune response [45]. In a similar approach, Kroll et al. developed B16-F10 melanoma membrane-wrapped PLGA NPs loaded with CpG oligodeoxynucleotide 1826 (CpG), a nucleic acid-based immunological adjuvant known to trigger APC maturation, and tested these in combination with a CTLA4 and anti-PD-1 checkpoint blockade cocktail [75]. The nanovehicles simultaneously delivered syngeneic cancer antigens with a powerful immunological adjuvant to promote antigen presentation. When applied separately in vivo, the CpG-CCNPs or checkpoint blockade cocktail did not significantly impair the growth of B16-F10 tumors. However, when combined, the systems synergistically promoted a strong antitumor response and modulated various aspects of the immune system [75]. Together, both of these studies indicate the potential for CCNPs to trigger superior anti-cancer immune responses by enabling codelivery of tumor antigens and adjuvants.

As the field matures, researchers will continue to design new ways to enhance immunotherapy mediated by CCNPs. Yang et al. recently showed that CCNP delivery to dendritic cells (DCs) could be enhanced by modifying CCNPs with mannose (which binds receptors on DCs) through lipid anchors [64]. The CCNPs consisted of PLGA cores encapsulating R837, an agonist against toll-like receptor 7 (TLR-7), wrapped with B16-OVA melanoma cancer cells and functionalized with mannose. The mannose-modified nanovaccine exhibited impressive DC uptake, triggering DC maturation. It also successfully traveled to draining lymph nodes post-transdermal injection and facilitated potent tumor-specific immune responses [64]. In another unique nanovaccine design, melanoma cell membrane fractions were coated onto PLGA NPs and their ability to effect fibroblast-mediated invasion, change experimental metastasis, and induce an immune response in immunocompetent mice was evaluated [43]. The nanovaccine successfully inhibited cancer cell migration toward fibroblasts, significantly decreased metastatic burden, and increased cytotoxic T lymphocytes, indicating membrane-wrapped nanovaccines not only show potential as antigen delivery vehicles for primary tumor elimination, but also as metastasis inhibitors. 

In summary, CCNPs have great potential as either prophylactic vaccines to protect patients from tumor cell challenges or as therapeutic agents to shrink tumors by inducing anti-cancer immune responses. It is observed that tumor antigen presentation from the membrane coating alone, even in highly immunogenic contexts, may not be powerful enough to overcome the immunosuppressive tumor microenvironment. Therefore, technologies must be combined with adjuvants, immune checkpoint blockade therapies, or other approaches to achieve optimal anti-cancer effects. Nevertheless, the biocompatible nature of CCNPs makes them promising as personalized therapies to induce cancer-specific immune responses for individual cancer patients. 

## 4. Challenges and Path Forward

While many of the described successes of CCNP systems are extremely encouraging, there are still many challenges to address before these technologies become commercially available. One potential issue is the need for patient education, as concern may develop over having cancer cell-derived material injected into the body. Although patients who already have cancer may be willing to overlook this concern if it provides a chance to eradicate their disease, healthy patients who are at-risk for certain types of cancer and wish to use this technology as a preventative vaccine may be less receptive. In addition to educating the population, stringent testing and procedures will have to be developed to ensure that the membrane coatings are pure (lacking any internal component of the source cells) and do not contain any molecules that might promote cancer growth. As the development of membrane-wrapped NPs is already becoming more mature, with proper tests this should not be an issue and this hurdle could be feasibly overcome. 

One of the biggest draws of using cell membranes to coat NPs is the ability to have a personalized treatment. NPs coated with membranes derived from a patient’s own cells should be able to evade unwanted immune responses that can occur when foreign material is introduced to the body. However, the feasibility of creating CCNPs for each individual patient is a significant question. Preparing patient-specific CCNPs will require strict quality control and regulatory methods. Additionally, while biopsy samples could be used to create CCNPs for patients with existing tumors, the production of CCNPs for prophylactic vaccination will require a different approach. Donor cells could possibly be used, but immunostimulatory issues may arise. One way this could be mitigated is by preparing particles with mixed membrane coatings, as several studies have shown this can imbue the NPs with properties of both membranes [57,68,111]. In this case, donor cancer cell membranes could be mixed with RBC or platelet membranes from the patient, lessening the portion of foreign membrane material to minimize an immune response. This strategy needs to be explored in many models in order to validate its use. An additional consideration for the use of CCNPs in a prophylactic setting is the need to define the appropriate patient population based on genetic testing or family history of specific cancers. However, if the concerns mentioned here can be sufficiently addressed, the potential impact of CCNPs for personalized cancer therapy is vast. 

Lastly, for CCNPs to be successful in the clinic, methods for manufacturing scale-up need to be developed. Particle replication on a small laboratory scale is already difficult due to the complex biological components involved and concerns of batch to batch consistency need to be addressed. One of the largest hurdles in scaling up the process is the need for large quantities of membranes. This requires millions, if not billions, of cells, and the facilities to grow them. Besides producing the necessary amount of membrane material, the scaling up of the NP cores is another concern, especially if the design is more complex or a multi-component system. The more complicated a nanotherapy fabrication is, the more difficult it is to create reproducible and identical particles at large scale. Finally, the assembly of how membrane vesicles are fused with NP cores could be difficult to replicate at a large scale. In short, the more steps to the process, the more difficult it will be to produce commercial quantities of high-quality material. This must be addressed before membrane-wrapped NP technologies can reach clinical trials or achieve FDA approval.

## 5. Conclusions

This review has highlighted the current state-of-the-art in developing CCNPs for the management of cancer. CCNPs have immense potential as tools to improve the imaging and treatment of tumors, but they also face substantial challenges in translating to the clinic. The future of the field lies in solving these issues or working around them to deliver specific, personalized therapy to single patients. While difficult to accomplish, the chance of eradicating even a single type of cancer, whether by therapy or vaccine, will continue to drive the many paths of research surrounding CCNPs described in this Review.

## Figures and Tables

**Table 1:** Breakdown of cancer cell membrane-wrapped nanovehicles mentioned in the text and their purposes.

Membrane Source	Core NP Material	Cargo Loaded	Particle Purpose (Besides Homotypic Targeting)	Year	Ref.
4T1	poly(caprolactone); Pluronic F-68	paclitaxel	drug delivery	2016	[40]
4T1	gold nanocages	doxorubicin	PTT; hyperthermia-triggered drug release	2017	[42]
4T1	poly(cyclopentadithiophene-*alt*-benzothiadiazole)		PTT; PDT; PA imaging	2018	[61]
4T1	PCN-224	tirapazamine	PDT; drug delivery	2017	[73]
MDA-MB-435	Ln-doped upconversion nanocrystal		FL imaging	2016	[54]
MDA-MB-435	PLGA	DiD fluorophore	FL imaging	2014	[45]
Luciferase- expressing MDA-MB-231	PLGA		FL imaging	2019	[43]
MCF-7 ^1^	PLGA	indocyanine green	PTT; PA/FL imaging	2016	[74]
MCF-7 ^1^	PLGA	doxorubicin; hemoglobin	PDT; drug delivery	2017	[63]
MCF-7 ^2^	melanin		PTT; PA imaging	2019	[68]
B16-F10 ^2^	hollow copper sulfide	doxorubicin	drug delivery	2018	[57]
B16-F10	mesoporous silica	glucose oxidase	immunotherapy; starvation therapy	2019	[55]
B16-F10	hollow manganese dioxide	chlorin e6; glucose oxidase	PDT; starvation therapy	2019	[67]
B16-F10	PLGA	CpG 1826	Immunotherapy ^4^	2017	[75]
B16-F10	PLGA	monophosphoryl lipid A	Immunotherapy ^4^	2014	[45]
B16-OVA ^3^	PLGA	imiquimod	Immunotherapy ^4^	2018	[64]
HeLa	iron oxide	doxorubicin	drug delivery	2016	[44]
HeLa	PLGA	doxorubicin; siRNA	drug delivery	2019	[46]
HeLa		doxorubicin; indocyanine green	PTT; drug delivery (carrier free)	2018	[76]
HepG2	PLGA	doxorubicin	drug delivery	2019	[62]
H22	iron oxide	doxorubicin	drug delivery	2016	[44]
SMMC-7721	superparamagnetic iron oxide	chlorin e6	PDT; MR/NIR imaging	2018	[72]
UM-SCC-7	iron oxide	doxorubicin	drug delivery	2016	[44]
CAL 27	Ln-doped upconversion nanocrystal		FL imaging	2016	[54]
LNCaP-Al	mesoporous silica	doxorubicin; calcium carbonate	drug delivery; pH sensitive release	2019	[56]
DU 145	Ln-doped upconversion nanocrystal		FL imaging	2016	[54]
U87	PLGA		Immunotherapy ^4^	2019	[43]
HCT 116	Ln-doped upconversion nanocrystal		FL imaging	2016	[54]

^1^ Membranes were mixed with PEGylated phospholipid (DSPE-PEG) before coating; ^2^ Membranes were mixed with red blood cell membranes before coating; ^3^ Membranes were modified with mannose after coating; ^4^ Particles were not used for homotypic targeting; Note: Murine mammary (4T1), human mammary (MDA-MB-435, MDA-MB-231, MCF-7), murine melanoma (B16-F10, B16-OVA), human cervical (HeLa), human hepatocellular (HepG2, H22, SMMC-7721), human squamous (UM-SCC-7, CAL 27), human prostate (LNCaP-Al, DU 145), human glioma (U87), human colorectal (HCT 116).
